# Rigid proteins and softening of biological membranes—with application to HIV-induced cell membrane softening

**DOI:** 10.1038/srep25412

**Published:** 2016-05-06

**Authors:** Himani Agrawal, Matthew Zelisko, Liping Liu, Pradeep Sharma

**Affiliations:** 1 Department of Mechanical Engineering, University of Houston, Houston TX, USA; 2 Department of Mathematics and Department of Mechanical Aerospace Engineering, Rutgers University, New Brunswick NJ, USA; 3 Department of Physics, University of Houston, Houston TX, USA.

## Abstract

A key step in the HIV-infection process is the fusion of the virion membrane with the target cell membrane and the concomitant transfer of the viral RNA. Experimental evidence suggests that the fusion is preceded by considerable elastic softening of the cell membranes due to the insertion of fusion peptide in the membrane. What are the mechanisms underpinning the elastic softening of the membrane upon peptide insertion? A broader question may be posed: insertion of rigid proteins in soft membranes ought to stiffen the membranes not soften them. However, experimental observations perplexingly appear to show that rigid proteins may *either* soften or harden membranes even though conventional wisdom only suggests stiffening. In this work, we argue that regarding proteins as merely non-specific rigid inclusions is flawed, and each protein has a unique mechanical signature dictated by its specific interfacial coupling to the surrounding membrane. Predicated on this hypothesis, we have carried out atomistic simulations to investigate peptide-membrane interactions. Together with a continuum model, we reconcile contrasting experimental data in the literature including the case of HIV-fusion peptide induced softening. We conclude that the structural rearrangements of the lipids around the inclusions cause the softening or stiffening of the biological membranes.

Protein inclusions in cell membranes are vital for life sustaining functions of a cell. The cell membrane—sometimes idealized as a fluid membrane—is very soft and deformable^1^. The inclusions have a profound impact on the bending rigidity of the membrane^2^ and this in turn has considerable influence on its various biophysical functions. Several studies, both *in vitro* and *in silico*, have been performed to examine the effect of protein inclusions on the bending rigidity of membranes^2^,^3^,^4^,^5^,^6^,^7^,^8^,^9^,^10^,^11^,^12^,^13^. Mechanically, proteins are usually regarded as *rigid* inclusions. In most theoretical models, such a notion immediately suggests that a membrane will *stiffen* due to the presence of these rigid inclusions (—provided that the inclusions are anchored in the membrane and do not diffuse). However, this leads to some rather interesting paradoxes. As will be shown in the Theoretical Results Section, a conventional Helfrich-Hamiltonian based approach yields the result that the apparent bending modulus of a membrane in the presence of anchored rigid proteins is *κ*_*b*_/(1 − *f*), where *κ*_*b*_ is the bending modulus of the virgin membrane and *f* is the area fraction of the proteins. This naive result suggests that all proteins (which are essentially rigid compared with the membrane) will stiffen the membrane in an *identical* manner. In other words, there is no protein *specificity*. Experiments suggest otherwise. In the case of HIV, recent and pioneering experiments^7^,^8^,^9^ find that the addition of HIV-1 fusion peptides decreases the apparent bending modulus of the membrane. Other experimental results show exponential decrease of the bending modulus of a DOPC bilayer with linearly increasing mole fraction of Alamethicin^10^,^11^, an antibiotic peptide produced by a fungus. Similarly, another study shows progressive decrease of membrane bending with increasing melittin concentration^12^, a peptide which is an active component of bee venom. Conversely, tests have been performed on red blood cells (RBCs) infected by a parasite which introduces Ring-infected erythrocyte surface antigen (RESA), a protein produced by the parasite, into the membrane of the host blood cell. Compared to healthy RBCs, the stiffness of the infected RBCs was significantly *higher*^13^—hence hardening was observed. The aforementioned experiments are quite interesting but also perplexing in the sense that *both* hardening and softening are observed when proteins or inclusions (that are essentially rigid) impregnate a soft membrane.

Perhaps the most exhaustive experimental study pertaining to the theme of the current work is that by Nagle and co-workers^7^,^8^,^9^ on HIV-1 fusion peptide induced softening which inspired us to look deeper in to the question of softening and hardening of membranes by rigid proteins and peptides. Since we will use this example both in our molecular dynamics simulations as well in our continuum model, a few more details on this case are warranted. The surface of HIV is composed of a lipid membrane containing many proteins. One of these viral proteins is glycoprotein 41 (gp41), a transmembrane protein that extends out of the membrane into the ectodomain. This extended domain of gp41 is responsible for initiating contact between the virion and the target cell. The virus then enters the target cell via receptor mediated endocytosis, whose mechanics is described^14^,^15^. The N-terminal of gp41 is preceded by an apolar segment generally referred to as the fusion domain or fusion peptide (FP). Following other works, here the fusion peptide is taken as the last 23 amino acids of gp41 leading up to and including the N-terminal; this section is called FP23^16^. The FP23 interacts with the target cell membrane in a non-specific way causing thinning (Fig. 1) and, in turn, softening of the membrane which subsequently allows pore formation. Through this pore, HIV can release RNA into the target cell for replication^8^. Using diffuse X-ray scattering, Nagle and co-workers^7^,^8^,^9^ discovered that the bending modulus of a membrane decreases upon addition of HIV fusion peptide. Depending on the composition of the membrane, they found that the bending modulus may be reduced to between 9 to 35% of the original value.

In this work, we propose that membrane proteins cannot be regarded as inert mechanically rigid objects that bond coherently with the ambient lipids. Specificity must be built into the models that purport to describe their effect on membrane mechanical properties. We hypothesize that each protein (rigid as it may be) has a unique mechanical signature parametrized by its specific interfacial coupling to the surrounding membrane. Together with this hypothesis, all-atom molecular dynamics (MD) simulations, and a complementary continuum mechanics model, we attempt to address the questions raised in the preceding paragraphs.

## Results

### Molecular dynamics simulations on HIV-1 fusion peptide and DMPC system

To better inform the development of a continuum model (see Theoretical Results and Theoretical Formulation in the Methods Section) we performed all-atom MD calculations to assess the changes in the elastic properties of a prototypical membrane due to the addition of HIV fusion peptide. Quantitative estimates of the overall membrane bending modulus were obtained in the absence and presence of proteins with a mole fraction of 0.01. Membrane properties close to the protein-membrane interface were also investigated to obtain insights into membrane softening mechanism.

**Figure 2:**
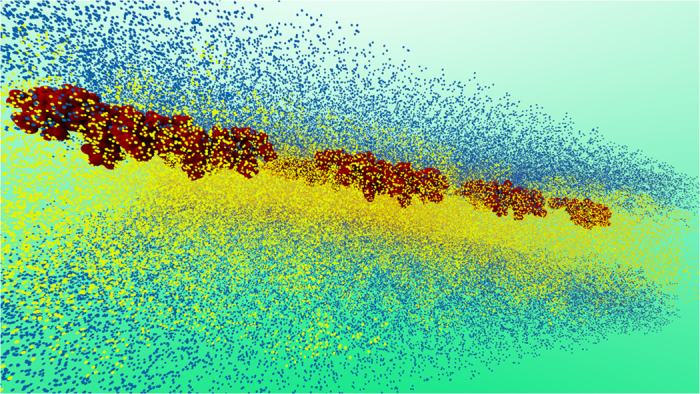
Large Scale all-atom MD simulations were performed on a system of 1600 lipids with 16 HIV FP23. The lipids are represented as yellow dots while the proteins are represented as red “blobs”. The size of the simulation box is 22.35 nm × 22.35 nm surrounded by water molecules—which are shown in blue.

Two separate simulations are performed. The first, using a pure DMPC bilayer as the control, and the second using the same bilayer with 0.01 mole fraction of inserted FP23. This mole fraction is consistent with the amounts used in experimental work, and results in 16 peptides inserted into the simulated bilayer^9^. The pure DMPC bilayer contains 1600 lipids, 800 in each leaflet, and equates to a 21.92 nm × 21.92 nm patch (Fig. 2). With the FP23 inserted, the patch size increases to 22.35 nm × 22.35 nm. Each case was energy minimized and then equilibrated using an NPT ensemble for 10 ns to ensure that there were no artifacts left from the creation of each topology. After equilibration was completed, a production simulation was performed for 10 ns for each case.

Experimentally, a common approach to estimate the bending modulus of a membrane is via recourse to so-called flicker spectroscopy which involves measurement of the thermal fluctuation spectra of the membrane^17^. Closed-form statistical mechanics formulae exist that then provide the required link between the thermal fluctuation measurements and the bending modulus. The same approach is also used in molecular dynamics simulations and simply involves tracking the atomic coordinates to measure the appropriate fluctuations^18^. However, this poses some difficulty for the cases presented here. First, the simulation “box” needs to be very large. This is necessary for capturing the necessary long wavelength fluctuations associated with the bending energy of the membrane and requires the length and width of the membrane to be greater than some critical length (around 20 nm). The lipid bilayers, with and without FP23, used here are large enough to capture these long wavelength fluctuations. Another problematic condition is that the simulations need to be run for very long times to achieve a good ensemble average of the fluctuation spectrum. Typically, this means that a simulation will need to be run for at least 1 microsecond but preferably longer. The aforementioned difficulties have the following implications—given the current available computing power, we can only use the fluctuations-based approach to compute the bending modulus if we use coarse-grained molecular dynamics. As will become evident in due course, our central idea necessitates a somewhat careful handling of the protein-lipid interface and accordingly the use of all-atom MD (instead of coarse-grained MD) is essential. Unfortunately, an all-atom MD simulation is computationally prohibitive in the present context. In the following we describe an approach based on some recent developments that allow us to extract the bending modulus in a computationally expedient manner—but with the fidelity of an all-atom simulation.

Recognizing and taking advantage of the fact that the bending modulus of a membrane and its lipid tilt and splay are closely related^19^,^20^,^21^, we will compute effective properties of the membrane by calculating the lipid splay^19^,^20^,^21^, both in the presence and absence of proteins. For a membrane (with or without protein inclusions) the bending modulus of a monolayer (*κ*_*m*_) is equal to the splay modulus of its constituent lipids (*χ*_*LL*_). In order to determine splay modulus, we defined vectors for lipid directors and normals to the outer surface. The tilt angle is the angle between the lipid director and surface normal at that point and splay angle is the angle between lipid directors. Splay angles were found between different combinations of lipid pairs. The number of lipid combinations were constrained by a convergence study in which we evaluated the impact of nearest neighbor distance on splay modulus. Based on this study, the nearest neighbor distance was chosen to be 8 nm. The methodology of finding bending modulus from tilt and splay is further detailed in the Methods Section.

**Figure 3:**
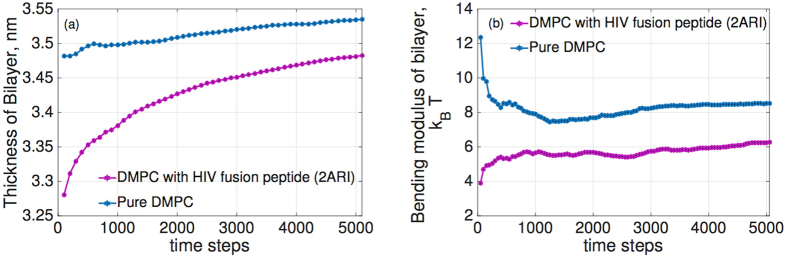
Thickness and bending modulus of the membrane as a function of the MD simulation time-steps. Only converged values are used for comparison. We note that the converged values clearly show decrease in thickness and bending modulus upon addition of HIV FP23 to the virgin lipid membrane.

**Figure 4:**
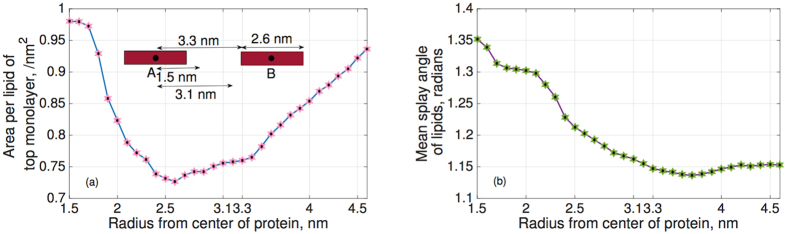
(**a**) The two red bars represent two FP23 separated by a distance of 4.6 nm from their centers. The plot shows decrease in the mean area per lipid as we move away from the center of protein, which again increases as we approach the other protein, (**b**) The mean splay angle reduces while moving away from protein, which indicates lessening of the impact of proteins on the surrounding lipids. Due to the decrease of splay, they occupy less area.

We find *P*(*α*), the normal probability distribution function of the splay angles at different time steps and then, the two molecule potential of mean force (PMF), which is defined as 
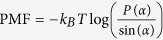
. The splay modulus is then estimated by performing a quadratic fit in the region of small *α* to the function *PMF*(*α*) (see Methods section). If the lipid system consists of only one type of lipids, the splay modulus of the monolayer is proportional to its bending modulus. The bilayer bending modulus is then twice of the splay modulus of lipid-lipid pairs. (Fig. 3) shows the final results in which we plot “thickness of membrane” and its “bending modulus” as the simulations evolve. The simulation results clearly show softening and thinning of the membrane upon addition of FP23, consistent with what was observed experimentally^7^,^8^,^9^. It is now well established that addition of FP23 to a pure bilayer will cause simultaneous softening and thinning as shown in (Fig. 3); the next question to answer is why does this happen? To expound on the mechanism behind the change in membrane properties we calculated the time averaged mean area per lipid and mean splay angles of lipids near the protein (FP23), shown in (Fig. 4). These quantities were measured as a function of distance from the center of mass of a FP23 inclusion. Data was used from every 100th time step of the simulation and a mean was taken for all lipids enclosed within a circle of a certain radius, with the FP23 centroid as the center of this circle. The figures evidently show how the area per lipid and the mean splay angle of the lipids change with the distance from the protein inclusion. Clearly, the lipids in the vicinity of FP23 are strongly impacted and there is a transition distance before the lipids assume an unaffected state similar to the pure membrane. From (Fig. 4), it is worth pointing out that the vales of mean area per lipid and mean splay angles are not equal at 1.5 nm and 3.1 nm, in spite of being at a same relative position with respect to proteins, albeit different proteins; the reason is that the mean is taken for all lipids that are enclosed by circle whose center is A and radii, 1.5 nm and 3.1 nm. The mean taken in this manner will be certainly different for 1.5 nm and 3.1 nm. Only if we chose the corresponding centers to be A and B for 1.5 nm and 3.1 nm respectively, the mean of area per lipid and mean splay angle will be the same. This insight allows us to frame the basic postulates needed for a theoretical model which is presented in the next section.

### Theoretical model and comparison with experimental predictions

The Helfrich-Canham elastic energy of the membrane^22^ (assuming protein to be rigid) given by:


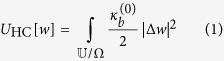


where 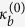 is the bending modulus of the membrane, 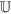 is the total region enclosed by the membrane protein system and Ω is the region occupied only be the petide. We have assumed that the lateral tension is negligible. A bilayer may have an asymmetric character if spontaneous curvature is present (–perhaps due to an asymmetric arrangements of proteins or peptides). However, the bending modulus is to the spontaneous curvature and is therefore ignored in what follows.

**Figure 5:**
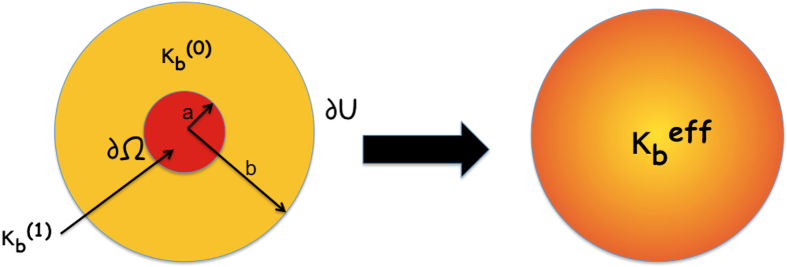
The left figure shows a peptide or protein of radius *a* and bending modulus 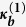 embedded in a membrane of radius *b* and bending modulus 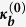. The outer boundary of the membrane is represented by 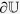 and the inner boundary between peptide and membrane by ∂Ω. We use an homogenization approach to obtain the effective bending modulus of this protein-membrane system, represented by 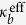.

In the naive case where we simply treat proteins or peptides as rigid objects (without any consideration of the fact that proteins alter the lipid structure in their vicinity), we can easily obtain (using a homogenization approach) an estimate of the effective bending modulus (Fig. 5):


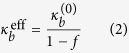


Here *f* is the area fraction of the included peptide. Further procedural details are provided in the Methods Section. From this formula, it is clear that the bending modulus derived from this naive classical model does not depend on the type of protein or membrane and merely on the concentration of peptide in the membrane. In other words, there is no protein-specificity. This is the reason why the existing models fail when it comes to explaining the softening of lipid bilayers due to FP23, Alamethicin, Magnanin and many others.

**Figure 6:**
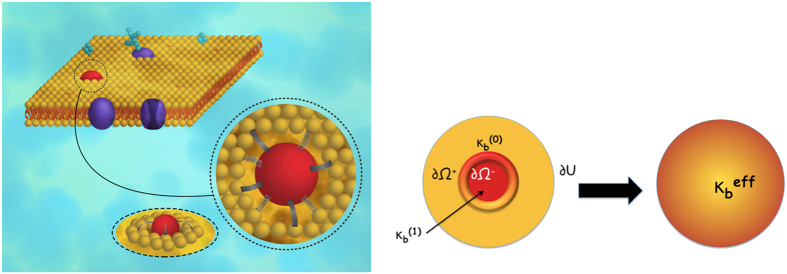
The central concept behind the theoretical model is the introduction of special interfacial boundary conditions represented by the parameters *k*_1_ and *k*_2_. The thin interface between protein and membrane is represented by solid bars and springs in the top figure. The bottom figure shows a peptide of radius *a* embedded in a membrane of radius *b*. The thin interface between peptide and lipid is also taken into account in this case. The outer surface of the membrane is represented by 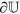. The inner surface of the protein is represented by ∂Ω^−^ and outer surface (which is towards the membrane) by ∂Ω^+^. The effective bending modulus of this protein membrane system is found by modifying the Helfrich Hamiltonian and taking into account the interface by introducing jump energies parametrized by *k*_1_ and *k*_2_.

To resolve this apparent paradox, we have developed a new theoretical model, which is based on the physical observations in the preceding section on atomistic simulations. We propose that at the molecular scale, the insertion of a protein in the membrane breaks the original in-plane lipid-lipid interactions and protein-water molecules (ambient medium) bonds and forms new bonds between protein and lipids (these are van der waals and electrostatic interactions between whole molecules, not actual bonds that are being broken). This causes structural rearrangement of lipids around the protein. So there is a thin interfacial layer between protein and lipid which has hybrid properties of lipids and proteins, as shown in (Fig. 6). It is this layer which leads to these anomalous observations. Based on these physical observations, and the fact that different proteins have different softening effects and in some cases even a stiffening effect, we postulate that the protein-membrane interface must play a role. The effects of this thin re-arranged layer of lipids may be captured by *not* adopting perfect boundary conditions typically assumed in theoretical models of the inclusion-matrix interface. We propose that the effects of this thin re-arranged layer of lipids may be captured by penalizing the jump of displacement and rotation angle in a Helfrich-type continuum model. We also propose that these jumps in displacement and rotation angle cost energy and should be added to Helfrich Hamiltonian in the following way:


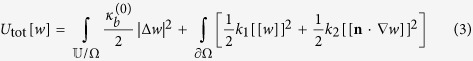


Where [[*w*]] and [[**n** ⋅ ∇*w*]] are jumps in displacement and rotation angle respectively, and *k*_1_, *k*_2_ > 0 are corresponding stiffness factors. Incorporating the new jump conditions, we can derive the following effective bending modulus (the detailed derivation is presented in the Theoretical Formulation of the Methods Section):


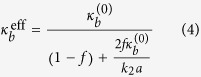


The above expression clearly indicates that value of 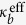 can be greater or less than 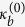 depending on the value of extra term 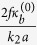 in the denominator. The value of *k*_2_ largely depends the type of protein- lipid system. Also, taking the limit *k*_2_ → ∞ in the above expression we readily recover the classic result 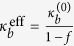.

**Figure 7:**
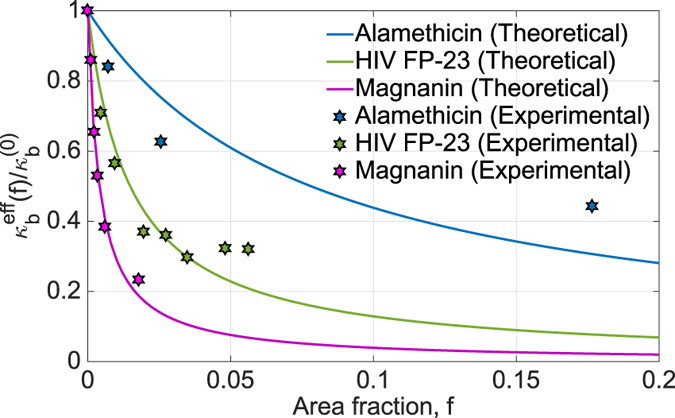
The plot shows effective bending modulus as a function of area fraction of peptide in a membrane for HIV FP23, Alamethicin and Magnanin. The solid lines represent the theoretical values of effective bending modulus corresponding to the area fraction *f*. The dotted points in corresponding colors are the experimentally observed values of the bending modulus corresponding to that *f* for that protein. Our theoretical predictions match well with that of experimental observations and explain the mechanics behind the softening process.

Experiments conducted by Nagle *et al*.^7^,^8^,^9^ show that the effective bending modulus of DOPC decreases as the mole fraction of fusion peptide (FP23) of gp41 of HIV-1 virus is increased. Similar softening is observed when either Alamethicin or Magnanin^3^,^23^ is added to a bilayer. Now, we fit the updated model to the experimental data, and the results are shown in (Fig. 7). Our modified model aligns well with the experiments. We also evaluate *k*_2_ for each protein membrane system in the plot which is shown in (Table 1).

In contrast to the softening cases mentioned in the preceding paragraph, RESA–a plasmodial protein, is one of the proteins that cause membrane stiffening. It plays a critical role in modulating the mechanical properties of RBC membranes. Impact on deformability at several stages after infection has been experimentally observed^24^ which shows up to two fold increase in bending modulus of RBC’s membrane. This membrane stiffening or decrease in membrane deformability has been linked with progression of the parasite infection. Although there is a lack of significant experimental data to make a comparison, it is clear that the model given in (4) can predict hardening of the membrane, 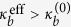, when the denominator is less than one.

## Discussion

We assume that in the absence of proteins, there is negligible splay in the membrane. Acknowledging that the mean thickness of membrane-protein system is less than that of membrane-only system (Fig. 3(b)), an implication is that the presence of proteins lead to a positive splay of lipids in its vicinity^25^. This suggests that in the vicinity of the membrane, the area per lipid should be higher and should decrease as we move away from the protein and should again increase as we approach the other protein—this also matches our interpretation from MD Simulations (Fig. 4(a)). We note that the change in tilt of lipids cannot change the area per lipid as the acyl chains elongate when they incline to maintain the constant lipid volume^25^.

The high area per lipid will lead to high splay in the vicinity of the protein (Fig. 4(b)). The increased splay angles, in turn, reduce the splay modulus and hence the bending modulus of the monolayer. To prevent water from entering into the hydrophobic regions of the membrane, the lower monolayer will follow the upper monolayer and hence an overall reduced bending modulus of the membrane in the presence of lipid inclusion ensues.

**Figure 8:**
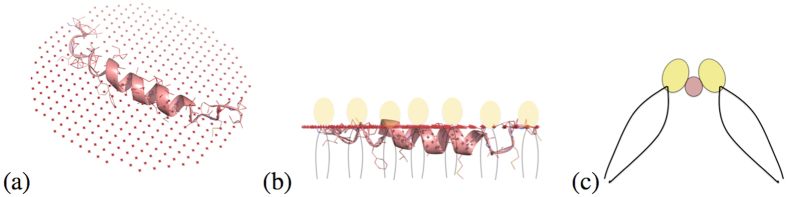
(**a,b**) The figure shows the spatial arrangement of FP23 with respect to the membrane. The red line is the hydrophobic-hydrophilic interface. The fusion peptide is almost parallel to the interface. Also, being mostly hydrophobic it lies with the hydrophobic tails of the bilayer, shown as black lines. (**c**) It shows the cross section of protein and lipids shown in (**b**). The lipids adjacent to the protein show positive splay.

In our theoretical model, we have postulated that there is structural rearrangement of lipids around the protein. This interpretation of our MD simulation explains that the rearrangement happens so as to match the increased area per lipid and increased splay angle requirements near the protein. This reduces the thickness of the membrane, which results in softening. The increase or decrease of thickness is decided by the structure of protein, or the hydrophobicity or hydrophilicity of its constituent amino acids. For example, HIV FP23 is highly hydrophobic and lies almost flat at the lipid hydrophobic-hydrophilic interface (explained in the Methods Section and Fig. 8(b)). Hence, for FP23, the lipids heads will stick to the top hydrophilic portions of the protein and will develop positive splay as shown in (Fig. 8(c)). It is these structural rearrangements which (from a continuum viewpoint) give rise to a jump in *w* and **n** ⋅ ∇*n* at the interface, and furthermore, different types of protein-lipid systems will result in distinctive jumps at the interface. Phenomenologically, these re-arrangements manifest in form of the newly introduced lipid-protein interface energy characterized by parameters *k*_1_ and *k*_2_.

While there has been considerable research on HIV vaccine development^26^,^27^, exploitation of membrane softening to design drugs has not been investigated. Conventional approaches behind tacking HIV protect against disease, not against infection and HIV remains latent in the body for a long period of time before the infection becomes apparent^28^. The real resiliency of HIV lies in its mutational and replicative abilities and adaptation to the human immune system^29^, which makes it highly difficult to curb HIV once the host is infected. Exploitation of the observed membrane softening for possible therapeutic purposes is an open question.

## Methods

### Molecular dynamics of HIV-1 fusion peptide in a lipid bilayer

All-atom MD Simulations were performed on a membrane-protein system using the freely available GROMACS software, version 4.5.6^30^. All simulations used the CHARMM-36 all atom force field which is optimized for biomolecules^31^, and the starting topologies were created using the Charmm-GUI membrane builder tool^32^. A leapfrog integration scheme was used, and all bonds were constrained allowing for a time-step of 2 ps to be used. For molecular dynamics simulations an NPT ensemble was used with semi-isotropic pressure coupling via the Parrinello-Rahman barostat method; the Nose-Hoover thermostat was used to maintain a temperature of 323 K during NPT equilibration and production MD.

#### Orientation of peptide in bilayer

FP23 structure (PDB id: 2ARI) was obtained from Orientations of Proteins in Membranes database (OPM) which provides spatial arrangements of membrane proteins with respect to the hydrocarbon core of the lipid bilayer^33^. The amino acid sequence of fusion peptide is detailed in^34^,^35^ and we find that most of the amino acids are hydrophobic. The orientation provided by OPM shows that the FP23 prefer to lay in a horizontal position, with respect to the bilayer normal, at the hydrophobic-hydrophilic interface of the outer surface of the lipid membrane.

#### Calculation of bending modulus from tilt and splay

**Figure 9:**
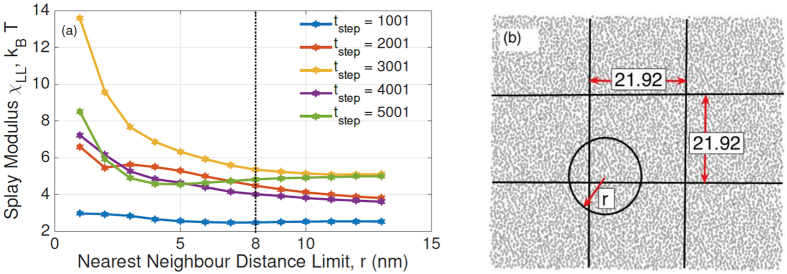
(**a**) A convergence study was performed to evaluate how far lipids can actually “feel” each other. Quantitatively, we compute a parameter called ‘splay modulus’ which is the energy penalty due to the splay distortion of the lipid molecules. This splay distortion costs more energy when the lipids are closer than when they are far away. Hence we need to chose an optimum cut-off distance beyond which the lipid pairs contribute minimally to the energy. It is the point at which the splay modulus saturates, and we find it to be 8 nm, (**b**) The figure shows 8 periodic images of the membrane-protein system, which is the middle box, surrounded by the 8 images. We accounted for influence of these images on the computation of splay modulus of the system. The quantity ‘r’ in the figure is the nearest neighbor cut-off distance (8 nm).

For a homogeneous membrane, the bending modulus of monolayer (*κ*_*m*_) is equal to the splay modulus of lipids (*χ*_*LL*_). In order to determine splay modulus, we need to define lipid directors and normals to outer surface. The lipid director is defined to be the vector connecting the midpoint between the head group phosphate atom, and the backbone C2 atom with the center of mass of the three terminal carbon atoms on the lipid tails^20^. The outer surface was generated by surface fitting of the points joining the midpoint between the head group phosphate atoms and the backbone C2 atom. The tilt angle is the angle between the lipid director and surface normal at that point and splay angle is the angle between lipid directors. Splay angles were found between different combinations of lipid pairs. The number of lipid combinations were constrained by a convergence study (Fig. 9(a)) in which we evaluated the impact of nearest neighbor distance on splay modulus. Based on this study, the nearest neighbor distance was chosen to be 8 nm. The periodic boundary conditions were also taken into account as shown in (Fig. 9(b)).

**Figure 10:**
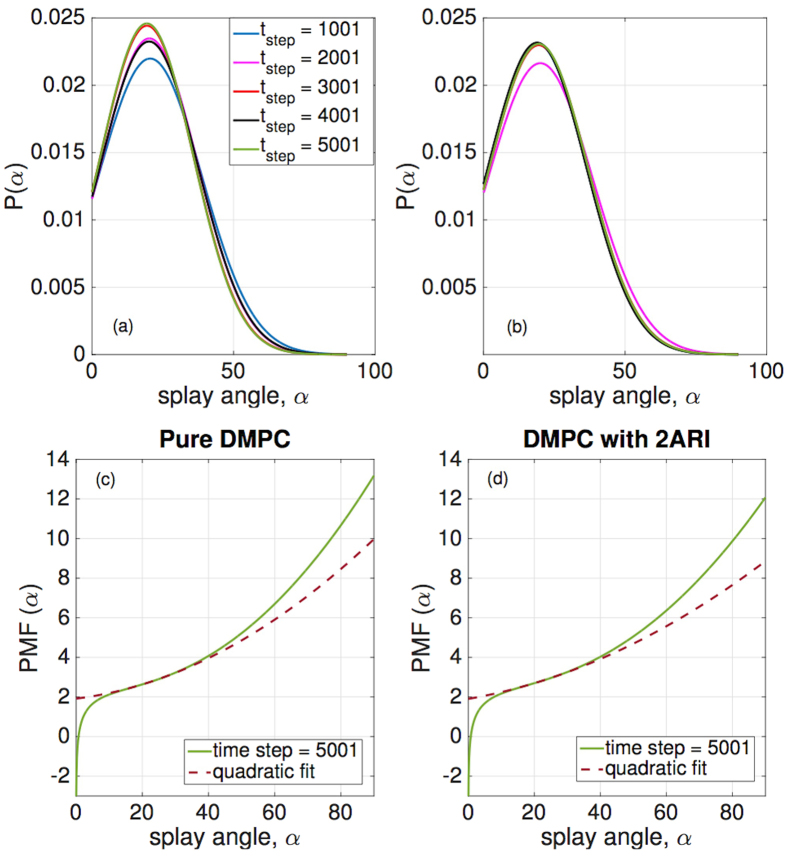
(**a,b**) Normalized probability densities *P*(*α*) of finding a pair of DMPC at an angle *α* with respect to each other. (**c,d**) The figure shows PMF (Potential of Mean Force) defined as 
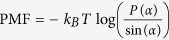
 where *α* are the angles between lipid-lipid pairs. A quadratic fit is performed to the PMF at small angles of *α* to evaluate Splay Modulus of the lipid monolayer.

We compute the normalized probability densities *P*(*α*) of finding a pair of DMPC at an angle *α* with respect to each other, shown in (Fig. 10(a,b)). The figures are drawn at different time steps and we can see that the probability densities are uniform across different time steps. The analysis was limited to nearest neighbors, and the lipid pairs which lie within 8 nm distance of each other were chosen. Among the chosen pairs, a pair was neglected if both the lipids have tilt angle more than 10 degree. Splay modulus for all the time steps was then obtained by performing a quadratic fit to the PMF plot as shown in (Fig. 10(c,d)). Splay modulus is twice the coefficient of the quadratic term in the PMF quadratic fit expression.

### Theoretical formulation for the modified problem

Let 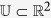 be an open bounded domain in the *xy*-plane. Consider a thin fluid membrane occupying 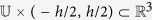, where *h* is the thickness of the membrane. If the thickness *h* compared to the area of U is very small, then the thin membrane may be idealized as a two-dimensional body; the thermodynamic state is described by the out-of-plane displacement 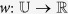.

At the outset we work in the Monge gauge and linearized setting. The Helfrich-Canham elastic energy of the membrane^22^ in the absence of lateral tension is given by:


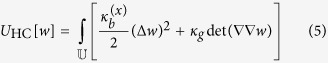


where *κ*_*b*_ and *κ*_*g*_ are the bending and Gaussian moduli, respectively, and 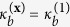 if **x** ∈ Ω; 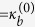 otherwise. The integral of Gaussian curvature over the membrane is independent of *R* and hence does not contribute to interaction energy^22^. Also, for the physically relevant case of rigid inclusions, the Gaussian modulus is irrelevant and hence forth, it is discarded. Future generalization of the work to finite elastic modulus of the inclusions must account for this.

To quantify the effects of insertion of protein in a bilayer and subsequent rearrangement of lipid molecules around it, we introduce a jump of displacement and rotation angle in the continuum model. Let the jump of a quantity (·) across ∂Ω be denoted by 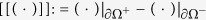, and hence the jump in displacement and rotation angle across the interface ∂Ω is denoted as [[*w*]] and [[**n** ⋅ ∇*w*]], respectively. Based on stability argument, the jumps in displacement and rotation angle shall be penalized by a positive internal energy, which to the leading order can be written as


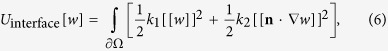


where *k*_1_, *k*_2_ > 0 are two phenomenological constants, and physically the above energy may be regarded as the protein-lipid interface energy. Summing up the effects of lipid-protein interaction, we propose the total elastic energy of the protein-bilipid membrane system as follows:


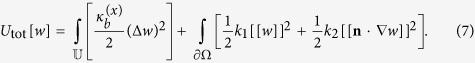


To mimic a uniformly bent homogeneous membrane we impose the following boundary conditions:


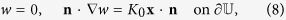


where *K*_0_ can be regarded as the macroscopic curvature of the membrane.

By the principle of minimum free energy, the equilibrium state of the membrane is determined by the variational principle


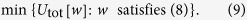


We now calculate the Euler-Lagrange equations and boundary conditions associated with the above variational principle (9). Let *w* satisfying (8) be a minimizer. Then for any perturbation *w* → *w* ± *εw*_1_ we have (0 < *ε* ≪ 1)


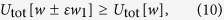


which implies


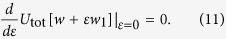


Upon integration by parts, the above equation can be rewritten as (*w*_1_ = ∇*w*_1_ = 0 on 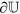)


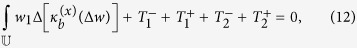


where 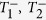 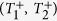 are integrals over the interior interface ∂Ω^−^ (exterior interface ∂Ω^+^) and given by


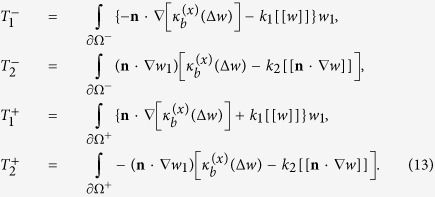


Since *w*_1_ and **n** ⋅ ∇*w*_1_ on ∂Ω^−^ and ∂Ω^+^ can be specified independently, by (12)–(13) we immediately obtain (14) as the Euler-Lagrange equations equations associated with the variational principle (9). It is typical that proteins are much more rigid than the bilipid membrane. Therefore, it will be useful to study the asymptotic behavior as 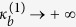. In this limit, to keep the total energy finite, we shall set Δ*w* = 0 on Ω. The third of (15) follows as one integrates the second of (14) over ∂Ω^−^. The rest of (15) is the same as in (14).


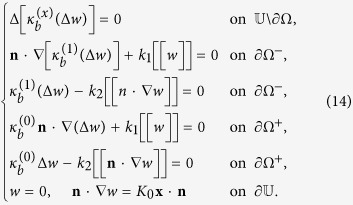


Moreover, it is typical that proteins are much more rigid than the membranes. Therefore, it is useful to study the asymptotic behavior as 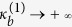. In this limit, the boundary value problem formed by (14) and (8) can be rewritten as


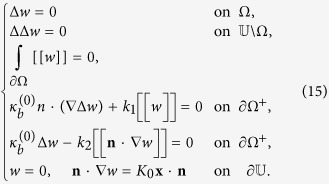


#### Solution of the homogenization problem

As shown above, the mid-plane profile 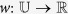 is determined by the variational problem (9); the associated boundary value problem is given by (14). In the limit that the inclusion is rigid, i.e, 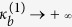, the boundary value problem can be rewritten as (15). If, in particular, both the inclusion Ω and the overall membrane 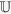 are circular and of radii *a* and *b*, respectively, by symmetry we infer the solution to (15) can be written as *w* = *w*(*r*). By (15)_1,2_ we can write the solution as


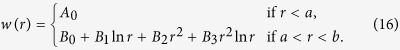


By (15)_3,4,5,6_, we have


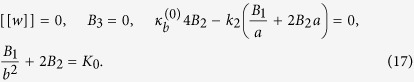


Solving the above set of equations we obtain,


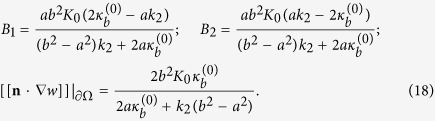


Inserting (16), (17) and (18) into (7), we obtain the total elastic energy of the system:


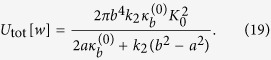


In the homogenization framework, the lipid-protein system is replaced by an equivalent homogeneous membrane with an effective bending modulus *κ*^eff^. With the same boundary conditions as in the last of, the homogeneous membrane admits the solution 
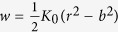
 and its total elastic energy is given by 
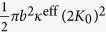
. Equating this energy to that of the lipid-protein system, i.e., (19), we identify the effective bending modulus as:


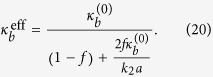


#### Material parameters and experiments

We have analyzed some key experimental results in the literature that show both softening and hardening upon addition of proteins in a bilayer. These experiments typically presents results for the effective bending modulus as a function of mole fraction of the proteins added. We have fit the experimental data to our theoretical model and estimated the corresponding value sof *k*_2_ using tleast square minimization curve fitting for that protein-bilayer system. We know that 
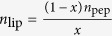
, where *x* is the mole fraction, *n*_pep_ is the number of peptide molecules, and *n*_lip_ is the number of lipid molecules. Also, 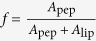, where *f* is the area fraction, *A*_pep_ is the representative total cross section area of peptides, and *A*_lip_ is the total cross section area of lipids which is equal to the area per lipid multiplied by *n*_lip_. A transmembrane protein can often have a complicated shape. We have idealized it as a cylinder of radius *a* along the entire thickness of the membrane and with the same volume as its original shape.


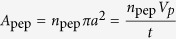


where *t* = thickness of bilayer, about 50 Å and *V*_*p*_ is the volume of peptide. Let 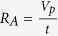, *R*_*L*_ = Area per lipid. Then,


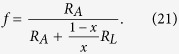


## Additional Information

**How to cite this article**: Agrawal, H. *et al*. Rigid proteins and softening of biological membranes–with application to HIV-induced cell membrane softening. *Sci. Rep*. **6**, 25412; doi: 10.1038/srep25412 (2016).

## Figures and Tables

**Figure 1:**
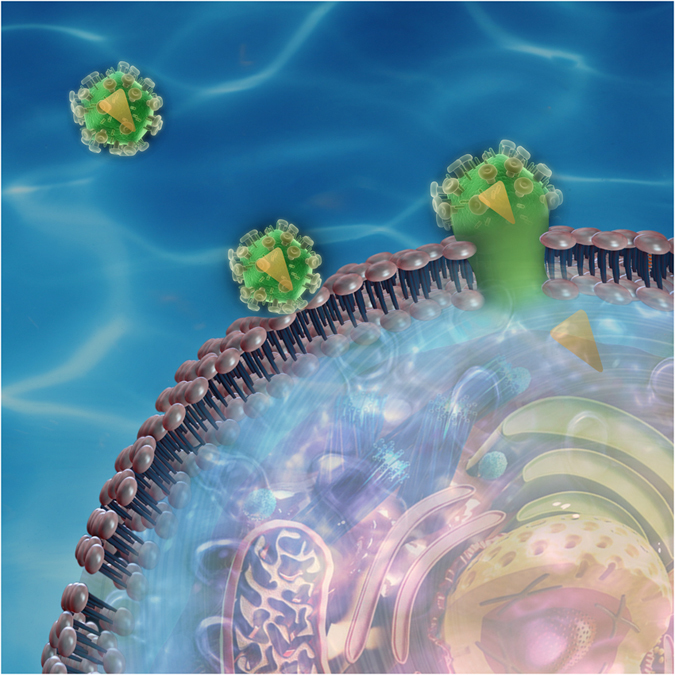
A schematic of the HIV Fusion Peptide softening a lipid bilayer membrane. The picture shows an HIV entering the host cell via membrane softening and thinning. Subsequently, through the creation of pore, the genetic material of HIV (shown in yellow) is injected into the host cell.

**Table 1:** The table lists the *k*_2_ values of various protein-membrane systems.

Protein, Membrane	*k*_2_ (*k*_*B*_*T*/*Å*)
Magnanin, POPC	0.03
Alamethicin, DOPC	0.28
FP-23, DOPC	0.13
